# Hypothermia enhances induction of protective protein metallothionein under ischemia

**DOI:** 10.1186/1742-2094-10-21

**Published:** 2013-02-04

**Authors:** Youn Hee Park, Young Mi Lee, Dong Sun Kim, Jaechan Park, Kyoungho Suk, Jong Kun Kim, Hyung Soo Han

**Affiliations:** 1 Department of Physiology, Kyungpook National University School of Medicine, 101 Dongin 2 Ga, Jung Gu, Daegu 700-422, Korea; 2 Anatomy, Kyungpook National University School of Medicine, 101 Dongin 2 Ga, Daegu, Jung Gu, 700-422, Korea; 3 Neurosurgery, Kyungpook National University School of Medicine, 101 Dongin 2 Ga, Daegu, Jung Gu, 700-422, Korea; 4 Pharmacology, Kyungpook National University School of Medicine, 101 Dongin 2 Ga, Daegu, Jung Gu, 700-422, Korea; 5 Emergency Medicine, Kyungpook National University School of Medicine, 101 Dongin 2 Ga, Jung Gu, Daegu 700-422, Korea

**Keywords:** Hypothermia, Ischemia, STAT3, MRE, Methylation, Gene expression

## Abstract

**Background:**

Hypothermic protection against ischemic stroke has been reported by many studies. Hypothermia is supposed to mitigate the effects of deleterious genes and proteins and promote the activity of protective genes and proteins in the ischemic brain. Metallothionein (MT)-1/2 is thought to be a crucial factor for metal homeostasis, immune function, and apoptosis. This protein was found to exert protective effects in models of brain injury as well. In the present study, we investigated the effect of hypothermia on MT expression and the underlying mechanisms.

**Methods:**

Cultured bEnd.3 brain endothelial cells were exposed to oxygen glucose deprivation and reperfusion (OGD+R). Reverse transcription PCR and western blot analyses were performed to measure the expression of MT, transcription factors, and methylation regulating factors. Transcription factor binding assays were also performed. Methylation profiles of the promoter area were obtained with pyrosequencing.

**Results:**

Hypothermia protected bEnd.3 cells from OGD+R. When the cells were exposed to OGD+R, MT expression was induced. Hypothermia augmented MT levels. While OGD+R-induced MT expression was mainly associated with metal regulatory transcription factor 1 (MTF-1), MT expression promoted by hypothermia was primarily mediated by the signal transducer and activator of transcription 3 (STAT3). Significantly increased STAT3 phosphorylation at Ser727 was observed with hypothermia, and JSI-124, a STAT-3 inhibitor, suppressed MT expression. The DNA demethylating drug 5-aza-2^′^-deoxycytidine (5-Aza) enhanced MT expression. Some of the CpG sites in the promoter MT=> it should be “the CpG sites in the MT promoter” showed different methylation profiles and some methylation regulating factors had different expressional profiles in the presence of OGD+R and hypothermia.

**Conclusions:**

We demonstrated that hypothermia is a potent inducer of MT gene transcription in brain endothelial cells, and enhanced MT expression might contribute to protection against ischemia. MT gene expression is induced by hypothermia mainly through the STAT3 pathway. DNA methylation may contribute to MT gene regulation under ischemic or hypothermic conditions.

## Background

Therapeutic hypothermia has been attracting attention as a method for treating ischemic stroke, traumatic and other types of brain injury, and cardiac arrest [1-5]. Hypothermia is believed to alter the expression of genes and proteins involved in cell growth, metabolism, immune responses, inflammation, cell death, and stress responses [6-8]. However many studies are still ongoing to clarify the underlying mechanisms.

Metallothionein (MT)-1/2 is a group of cysteine-rich and metal binding proteins that play roles in metal homeostasis, detoxification, and oxygen radical scavenging [9-11]. Accumulated data indicate that MTs play protective roles in brain injury models [12-14]. MT has been detected in ischemic brain [15,16], and our unpublished data showed increased MT expression in the brain vasculature of a stroke model under hypothermic conditions. Expression of MT genes is regulated at the transcriptional level. In the promoter area of MT genes, there are many binding sites for different transcription factors [17-20] such as metal response elements (MREs), glucocorticoid response elements (GREs), and signal transducer and activator of transcription (STAT). Metal transcription factor-1 (MTF-1) is one of the major factors regulating MT gene expression that is essential for transcription by metals including zinc, and mediates the responses to oxidative stress and hypoxia [21,22]. MTF-1 binds to MREs, which are present in six non-identical copies (MRE a through f) in the 5^′^-flanking region [23]. MTF-1 belongs to the zinc finger transcription factor family [24].

One study on STAT has shown that lipopolysaccharide (LPS)-induced activation of STAT1 and STAT3 binding leads to the activation of MT transcription [25]. STAT3 is continuously expressed in the central nervous system (CNS) and can be activated by a variety of pathological stimuli including oxidative stress, cerebral ischemic insults, and cytokines [26-29]. Studies demonstrated that methylation in the promoter region of the MT gene contributes to the regulation of the MT gene [30,31]. Expression of this gene is abrogated after treatment with 5-aza-2^′^-deoxycytidine (5-Aza), a compound that stimulates the removal of methyl groups from cytosines of the CpG island [30,31]. However the impact of MT methylation under ischemic or hypothermic conditions is unclear.

In the present study, we investigated the induction of MT gene expression in brain endothelial cells using an *in vitro* ischemia model involving oxygen glucose deprivation (OGD) and reperfusion. Further studies on transcriptional regulation of the MT gene with ischemia or hypothermia were also performed. Our goal was to elucidate the mechanisms of MT gene induction by hypothermia.

## Methods

### Cell culture

Immortalized mouse brain endothelial bEnd.3 cells were purchased from the American Type Culture Collection (Manassas, VA, USA). The cells were cultured with Dulbeco’s modified Eagle’s medium (Sigma Chemical, St. Louis, MO, USA) containing 10% fetal bovine serum (Sigma Chemical) at 37°Χ in a humidified 5% CO_2_. The cells were treated continuously with various concentrations (0, 0.01, and 1 μM) of 5-Aza (Sigma Chemical) for 3 days. Cells were also treated with various concentrations (0, 0.1, 1, and 10 μM) of JSI-124, a STAT3 inhibitor (Calbiochem, San Diego, CA, USA); 10 μM of U0126, an inhibitor of MEK1/2 (Cell Signaling, Beverly, MA, USA); or 1 μM of SB203580, a JNK inhibitor (Cell Signaling) for 30 minutes before OGD.

### Oxygen-glucose deprivation and reperfusion (OGD+R)

Cell cultures were subjected to ischemia-like injury through OGD for 4 hours by placing cultures in an anaerobic chamber (Forma, Thermo Scientific, Asheville, NC, USA) with an atmosphere of O_2_ tension less than 0.2% (5% CO_2_, 5% H_2_, and 90% N_2_) in a deoxygenated glucose-free balanced salt solution (BSS0). After 4 hours of OGD, cultures were reperfused by adding 5.5 mM glucose to the media at normoxia. Control cultures (no injury) were incubated with a balanced salt solution containing 5.5 mM glucose (BSS5.5). Cultures were placed in a humidified 37°C or 33°C incubator depending on experimental conditions. The cultured cells and media were harvested at different time points after reperfusion initiation.

### Cell viability assay

To measure cell viability, a non-radioactive cytotoxicity assay kit (Promega, Madison, WI, USA) was used to detect released lactate dehydrogenase (LDH) in the culture media. Briefly, 50 μL of the test samples were mixed with 50 μL of reaction mixture provided by manufacturer and incubated for 30 minutes at 25°C while protected from the light. The absorbance was measured at 490 nm using a GENius Plus microplate reader (Tecan, Männedorf, Switzerland). All samples were run in triplicate.

### Reverse transcription PCR

Total RNA was isolated from the cells using Trizol reagent (Life Technologies, Rockville, MD, USA) following the manufacturer’s instructions. For each sample, 2 μg of total RNA was reverse-transcribed into cDNA with Oligo(dT)_15_ primer and M-MLB reverse transcriptase (Promega) for 90 minutes at 37°C. The products were amplified using Taq polymerase. β-actin was used as the internal standard. The sample was heated to 94°C for 2 minutes followed by 27 cycles of denaturation at 94°C for 1 minute, annealing at 60°C for MT-1 or 55°C for MT-2 and β-actin for 1 minute, and extension at 72°C for 1 minute. Amplification was performed in a DNA thermal cycler (MJ Research, Watertown, MA, USA). The PCR products were separated in a 2% agarose gel containing ethidium bromide (Sigma Chemical). The bands were visualized and photographed using a gel documentation system (Bio-Rad**,** Hercules, CA, USA).

In addition to traditional RT-PCR, we also performed real-time PCR to quantify gene expression. Template cDNA was added to a PCR master mix containing SYBR Green (Roche, Manheim, Germany). The master mix contained HotStarTaq polymerase, which was included to avoid false positives in the PCR results. GAPDH was used as an internal control. Real-time quantification of gene expression was performed using a SYBR-Green real-time assay with a Roche cycler.

The PCR primer sets were as follows:

MT-I (159 bp) sense, 5^′^-ACCTCCTGCAAGAAGAGCTG-3^′^; antisense, 5^′^-GCTGGGTTGGTCCG ATACTA-3^′^

MT-II (209 bp) sense, 5^′^-CCGATCTCTCGTCGATCTTC-3^′^; antisense, 5^′^-ACTTGTCGGAAGCCTCTT TG-3^′^

β-actin (280 bp) sense, 5^′^-ATCCTGAAAGACCTCTATGC-3^′^; antisense, 5^′^-AACGCAGCTCAGTAACAGTC-3^′^

GAPDH (198bp) sense, 5^′^-AACAGCAACTCCCACTCTTC-3^′^; antisense, 5^′^-CCTCTCTTGCTCAGTGTCCT-3^′^

DNMT1 (240 bp) sense, 5^′^GCTCAAAGACTTGGAAAGA-3^′^; antisense, 5^′^-AACTGAAAGGGTGTCACTGT-3^′^

DNMT2 (250 bp) sense, 5^′^-CTGCACATGTGGTGG-3^′^; antisense, 5^′^-ACAGGCTTCTATTGTTTG-3^′^

DNMT3a (256 bp) sense, 5^′^-CTGGAGCTGCAAGAGTGTCTG-3^′^; antisense, 5^′^-ATGACCGGCACGCTCCACGAT-3^′^

DNMT3b (288 bp) sense, 5^′^-TTCGGCTTCCCTGCTCACTAC-3^′^; antisense, 5^′^-AGGCTCTGCTCCCACTGAGCA-3^′^

MBD1 (264 bp) sense, 5^′^-AGAGGAGTGGACAGCAGTCAC-3^′^; antisense, 5^′^-ATGTAGCCTTGGCAACCAGGC-3^′^

MBD2 (294 bp) sense, 5^′^-GTCCAGGTAGCAATGACGAGA-3^′^; antisense, 5^′^-GCCTCATCTCCACTGTCCATG-3^′^

MBD3 (320 bp) sense, 5^′^-CCATTACAGGCCAGCTCTCTG-3^′^; antisense, 5^′^-AGTCTGCAGCCCAGACTTGGG-3^′^

MBD4 (280 bp) sense, 5^′^-ATCGGACCTCAGGCAAGATGG-3^′^; antisense, 5^′^-GTCTTCAGGGTGCACCTGCTT-3^′^

MECP2 (240 bp) sense, 5^′^-TGAGCCTGAGAGCTCTGAGGA-3^′^; antisense, 5^′^-CACAGGCTCCTCTCTGTTTGG-3^′^

### Nuclear and cytosolic extraction and electrophoretic mobility shift assay

Nuclear extracts were isolated from the cultured cells. The extracts were prepared using a Nuclear Extract Kit (Active Motif, Carlsbad, CA, USA) according to the manufacturer’s instructions. The nuclear proteins were quantified with a Bio-Rad protein assay kit (Bio-Rad). The DNA-binding activity of MTF-1/MRE was measured with an electrophoretic mobility shift assay (EMSA) ‘Gel-Shift’ kit (Panomics, Redwood, CA, USA) according to the manufacturer’s instructions. The oligonucleotide representing MTF-1 was 5^′^-TT**TGCACTC**GTCTT**TGCACTC**GTC-3^′^ (core MTF-1 sequence is shown in bold, Panomics MTF-1/MREd EMSA Probe) and the oligonucleotide corresponding to MRE was 5^′^-CTC**TGCGCCC**GGCCC-3^′^ (core sequence is shown in bold, Panomics MREa EMSA Probe). Briefly, 1 μg of poly-(dI-dC) and 10 ng of biotin-labeled MRE probes were added to 5 μg of nuclear extract and incubated in binding buffer at 20°C for 30 minutes. The mixture was then separated in a non-denaturing polyacrylamide gel and subsequently transferred to Pall Biodyne B nylon membrane. After blocking, the membrane was incubated with streptavidin-horseradish peroxidase conjugate at room temperature for 15 minutes. Band detection was accomplished with 1X detection buffer provided with the kit. The shifted bands corresponding to the MTF-1 or MRE/DNA complexes were visualized relative to the unbound double-stranded DNA after exposing the membrane to X-ray film (Kodak, Toyko, Japan). For the competition experiments, unlabeled double-stranded DNA probe (cold probe) and mutated-DNA probe were used. Band density was analyzed with NIH Image J program (http://rsb.info.nih.gov/ij/).

### STAT3 binding assay

STAT3 levels in the cultured nuclear extract were measured using a TransAM STAT3 kit (Active Motif**)** to quantify transcription factor activation. This kit included a 96-well plate on which oligonucleotide containing the STAT3 consensus binding site (5^′^-TTCCCGGAA-3^′^) was immobilized. The active form of STAT3 contained in the nuclear extract specifically bound to this oligonucleotide. For this experiment, 5 μg of nuclear extract was used along with antibody directed against STAT3. A secondary anti-IgG antibody conjugated to horseradish peroxidase (HRP) was then added and absorbance was measure with a GENius Plus microplate reader (Tecan) at 450 nM with a reference wavelength of 655 nM. To evaluate the specificity of the assay, a wild-type consensus oligonucleotide was used as a competitor for STAT3 binding. This oligonucleotide prevented STAT3 from binding to the probe immobilized on the plate. Additionally, a mutated oligonucleotide was tested and predicted to have no effect on STAT3 binding.

### Western blot analysis

Cells were homogenized in lysis buffer (20 mM Tris–HCl pH 8.0, 137 mM NaCl, 10% glycerol, 2 mM EDTA, 1% Nonidet P-40, 1 mM Na_3_VO_4_, and 5 mM NaF). The lysates were centrifuged at 12,000 g for 20 minutes at 4°C (Bio-Rad). Proteins in the supernatant were quantified with a Bio-Rad protein assay kit (Bio-Rad). To monitor STAT3 phosphorylation, 30 μg of protein were boiled for 5 minutes at 100°C in gel loading buffer containing 20% mercaptoethanol and separated in 10% SDS-PAGE gels. The proteins were transferred to nitrocellulose membranes (Sigma Chemical) at 90 V for 90 minutes. The membranes were then blocked with 5% skim milk (Sigma Chemical) for 1 hour at room temperature and incubated overnight at 4°C with primary antibody (1:1000 dilution) against phosphorylated-STAT3 (Ser727) or total-STAT3 (Cell Signaling). After washing with PBS, the membranes were incubated for 1 hour at room temperature with HRP-conjugated secondary antibody (1:2000, Santa Cruz, CA, USA) and then washed again. Proteins in the membrane were visualized with an ECL system (Amersham, Piscatway, NJ, USA). The density of each band was analyzed with NIH Image J program (http://rsb.info.nih.gov/ij/).

### Methylation analysis with pyrosequencing

Genomic DNA was extracted with a Qiagen DNeasy kit (Qiagen Korea, Seoul, Korea) and treated with sodium bisulfite reagent. Briefly, 2 μg of genomic DNA was denatured by incubating with 2 M NaOH for 10 minutes at 37°C. Next, the denatured DNA was treated with freshly prepared 10 mM hydroquinone and 3 M sodium bisulfate (pH 5.0). The sample was amplified by PCR, and PCR products were cloned into a pGEM-T easy vector using the pGEM-T easy vector systems (Promega) according to the manufacturer’s protocol. Plasmid DNA was purified with a Wizard™ DNA purification system (Promega) and the DNA sequences of several independent clones were analyzed (Bionics, Seoul, Korea). Briefly, the PCR reactions were performed with one biotinylated PCR primer, which enabled the conversion of the PCR product into a single-stranded DNA template suitable for pyrosequencing. Confirmation of PCR product quality and freedom from contamination was established by separation in 2% agarose and ethidium bromide staining. Pyrosequencing was carried out using the PSQ96MA system (Biotage, Uppsala, Sweden) according to the manufacturer’s protocol and single-strand binding protein (PyroGold reagents). All primer sets were designed with the help of PSQ assay design software (Biotage) and the sequencing primers were manually checked for mis-priming sites. In addition, negative controls were run on the PSQ96MA instrument to check for background generated from the sequencing primers, biotinylated primers, or the template (MT-I F - GGGGAAAGTATTATAGGGATATG, R - Biotin-AAAACAACCTACCCTCTTTATAAT, S- GAAAGTATTATAGGGATATGATG).

### Overexpression and knockdown of the MT gene

We measured the protective effect of MT using an MT overexpression plasmid vector [14]. Briefly, full-length human MT-I cDNA (186 bp) originally cloned into a pDNR-LIB vector was purchased from Life Technologies Korea (Seoul, Korea). Human MT-I cDNA was amplified by PCR with primers containing *Bam*HI and *Xho*I restriction enzyme sites, digested with the restriction enzymes, and then ligated into a pcDNA3.1 plasmid containing a CMV promoter. This plasmid construct was sequenced at Solgent Co. (Daejeon, Korea) to verify the sequences. After transfection of bEnd.3 endothelial cells with the overexpression vectors (DNA concentration of 2, 10, and 20 μg in 4 μL of PolyMAG reagent (Magnetofection, Chemicell GMBH, Berlin, Germany)) for 48 hours, the increased MT expression was confirmed with RT-PCR and western blot analyses. The protective effect against OGD+R was measured using an LDH assay. MT-1 and MT-2 siRNA were purchased from Qiagen Korea. After treatment to bEnd.3 endothelial cells with siRNA targeting MT-1 (10 nM) or MT-2 (10 nM) in 3.5 μL of RNAiMAX reagent (Life Technologies Korea, Seoul, Korea) for 24 hours, the reduction of MT mRNA levels was confirmed with RT-PCR and the deleterious effect against hypothermia was measured using an LDH assay.

### Statistical analysis

All experiments were performed in triplicate or more. Data are presented as the mean ± S.E.M. Comparisons between groups were made using Sigma-Stat software (SPSS, Inc., Chicago, IL) and analyzed with a one-way ANOVA followed by *post hoc* comparisons. *P* values <0.05 were considered statistically significant.

## Results

### Hypothermic protection against ischemic insult by MT

**Figure 1:**
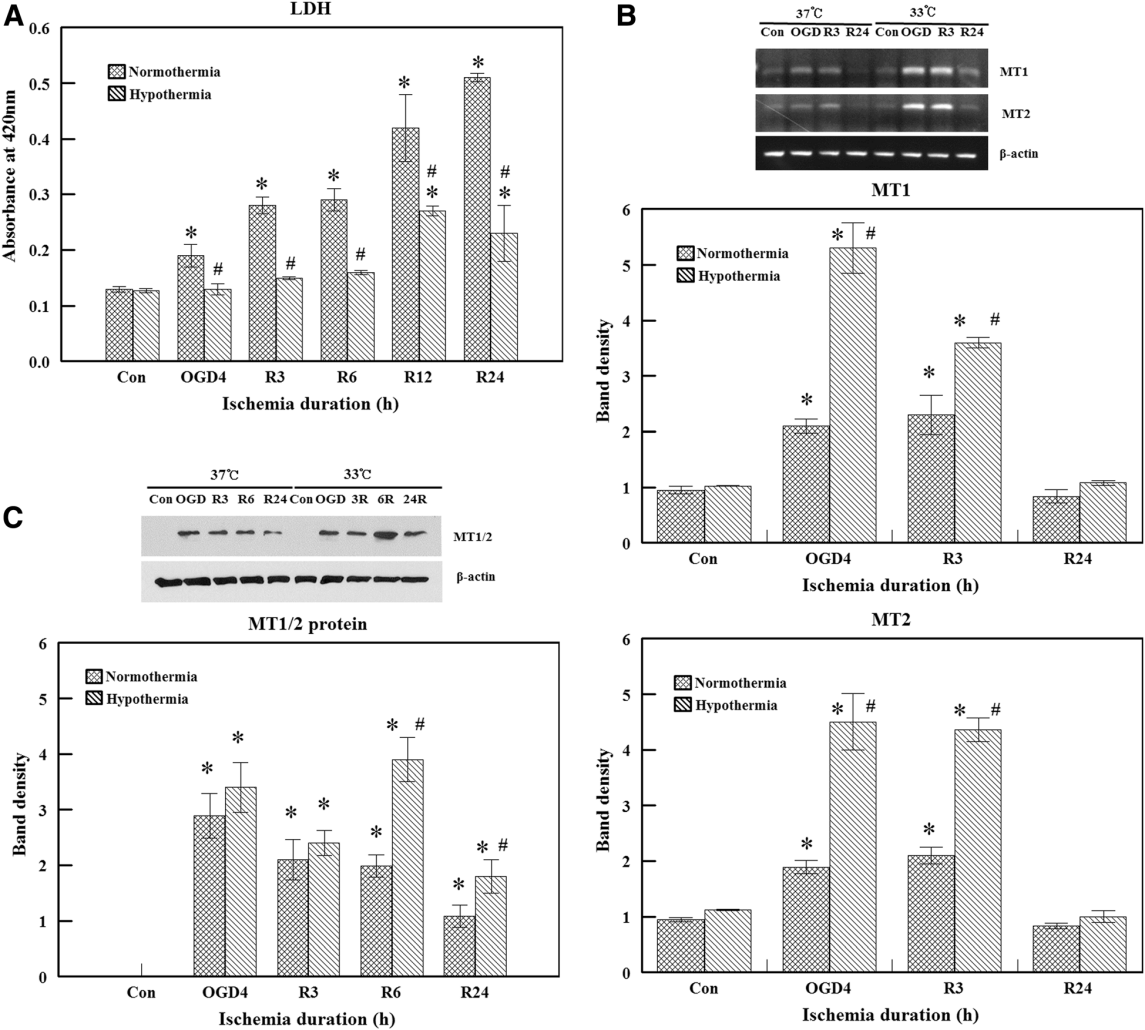
**Protective effect of hypothermia and induction of metallothionein (MT) gene expressions by hypothermia. (A) **Lactate dehydrogenase (LDH) activity in the culture media was measured after bEnd.3 cells were subjected to 4 hours of oxygen glucose deprivation (OGD) and 24 hours of reperfusion (R) at 37°C or 33°C. LDH levels increased by OGD+R were diminished by hypothermia. Experiments were performed in triplicate. **(B) **Total RNA was isolated from bEnd.3 cells cultured in the normal control state, after 4 hours of OGD, and 3 to 24 hours after reperfusion initiation at 37°C or 33°C. The induction of MT expression peaked after OGD and decreased to basal levels during reperfusion. Hypothermia-augmented MT expression compared to normothermia. β-actin was used as the internal control. Band densities of the MT gene are expressed as the mean ± S.E.M. of five experiments. *P <0.05 compared to the control; #*P *<0.05 compared to normothermia. **(C) **MT protein expression was measured by western blot analysis. MT protein expression was induced after OGD and slowly declined during reperfusion. The level was higher with hypothermia. Band densities are expressed as the mean ± S.E.M. of five experiments. *P <0.05 compared to the control; #*P *<0.05 compared to normothermia.

**Figure 2:**
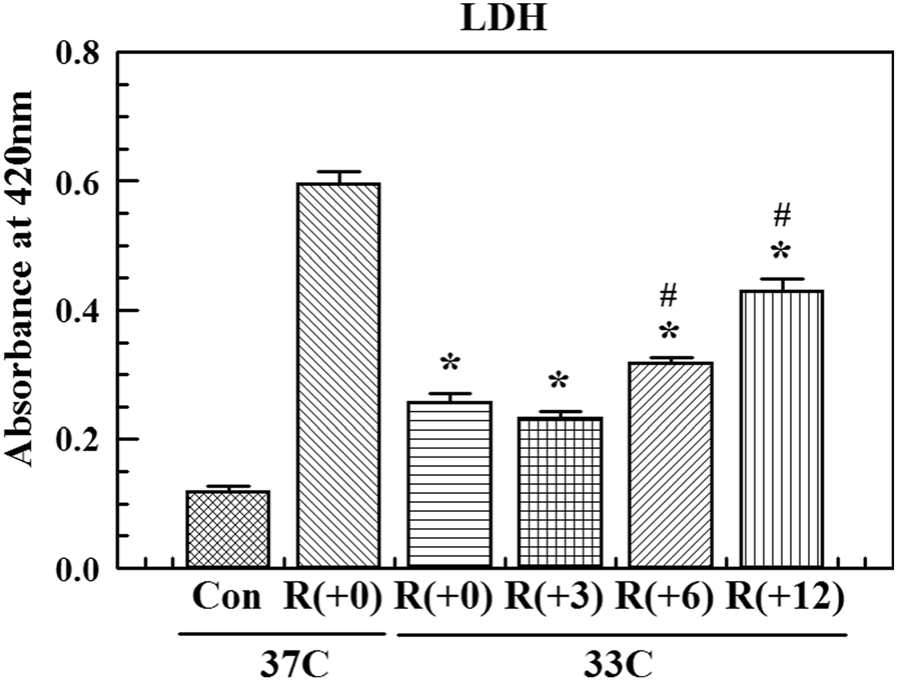
**Delayed protection by hypothermia. **We measured the effect of delayed hypothermia using an lactate dehydrogenase (LDH) assay. The protective effect of hypothermia was progressively reduced as the time of hypothermia initiation was delayed 3, 6, and 12 hours (R(+3), R(+6), and R(+12)). However, hypothermia still offered significant protection when the initiation of hypothermia was delayed up to 12 hours. Values represent the mean ± S.E.M. of three different experiments. *P <0.05 compared to 4 hours of oxygen glucose deprivation (OGD) and 24 hours of reperfusion (R + 0) at 37°C; #*P *<0.05 compared to 4 hours of OGD and 24 hours of reperfusion at 33°C.

To investigate the cytotoxic effect of OGD+R and protective effect of hypothermia, cellular injury was evaluated by measuring the levels of released LDH. At 37°C, LDH concentration increased after OGD+R. However, increases of LDH were not observed at 33°C even 6 hours after reperfusion (Figure 1A). These data indicate that hypothermia protected brain endothelial cells from ischemic insult. To evaluate the delayed effect, hypothermia was induced after the initiation of reperfusion. The protective effect of hypothermia was progressively reduced as the time of hypothermia initiation was delayed. However, hypothermia still offered significant protection when initiation was delayed up to 12 hours (Figure 2).

### Hypothermic enhancement of MT gene expression

**Figure 3:**
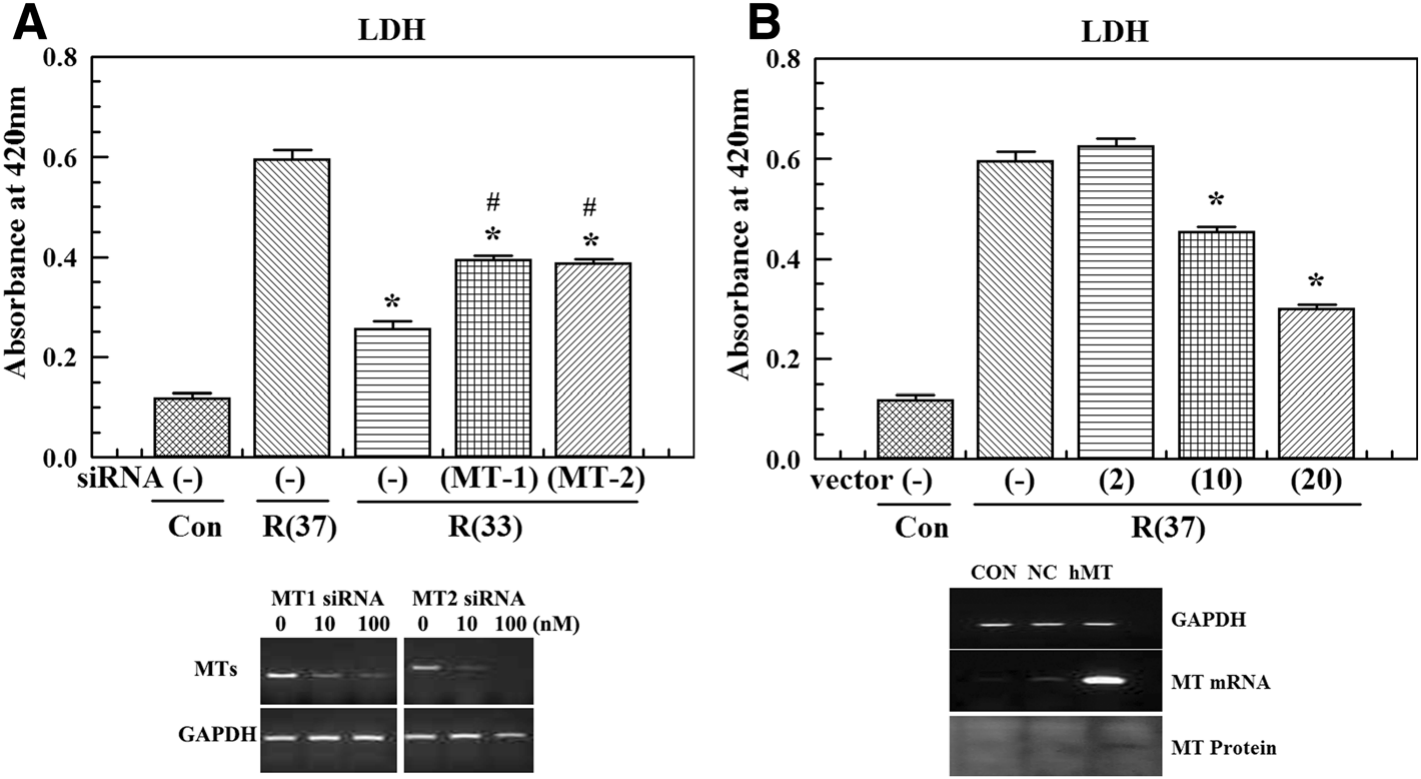
**Effects of metallothionein (MT) knock-down or overexpression on the cell death after ischemic insult. (A) **We measured the lactate dehydrogenase (LDH) levels in culture media harvested from cells treated with siRNAs (10 nM) targeting MT-1 or -2 after 4 hours of oxygen glucose deprivation (OGD) and 24 hours of reperfusion at 33°C. *P <0.05 compared to 4 hours of OGD and 24 hours of reperfusion without siRNA treatment at 37°C; #*P *<0.05 compared to 4 hours of OGD and 24 hours of reperfusion without siRNA treatment at 33°C. Suppression of MT gene expression by siRNA was confirmed by measuring the level mRNA using RT-PCR. GAPDH was used as an internal standard. **(B) **We measured LDH levels in culture media harvested from the cells treated with MT overexpression vectors (2, 10, and 20 μg) after 4 hours of OGD and 24 hours of reperfusion at 37°C. Values represent the mean ± S.E.M. of three different experiments. *P <0.05 compared to 4 hours of OGD and 24 hours of reperfusion without MT overexpression vector at 37°C. Overexpression of the MT gene by the vector-containing human MT-1 clone was confirmed by detecting the levels of mRNA (RT-PCR) and protein (western blot) in different conditions: Without MT-1 vector and transfection reagent (CON), transfection reagent only (NC), MT-1 vector in transfection reagent (hMT). GAPDH was used as an internal standard.

To determine the effect of hypothermia on MT gene expression, MT mRNA levels were measured with RT-PCR. MT gene levels with normothermia were increased after 4 hours of OGD and 3 hours of reperfusion. These levels returned to baseline after 24 hours of reperfusion. Hypothermia augmented the increased MT gene expression (Figure 1B). MT protein levels were measured by western blot analysis. Under normothermic conditions, increased MT protein levels were observed after 4 hours of OGD as well as 3 and 6 hours of reperfusion. After 24 hours of reperfusion, the protein levels of MT decreased. With hypothermia, MT protein levels were higher at 6 and 24 hours of reperfusion compared to the normothermic group (Figure 1C). We speculate that increased mRNA expression might have resulted in higher protein levels observed with hypothermia. To confirm the protective effect of MT against ischemic insult, we performed experiments using siRNA and an MT overexpression vector. Reduced induction of MT gene expression by siRNA attenuated the protective effect of hypothermia. In contrast, MT gene overexpression resulted in protection of endothelial cells against OGD insult with normothermia (Figure 3).

### DNA binding of MTF-1

**Figure 4:**
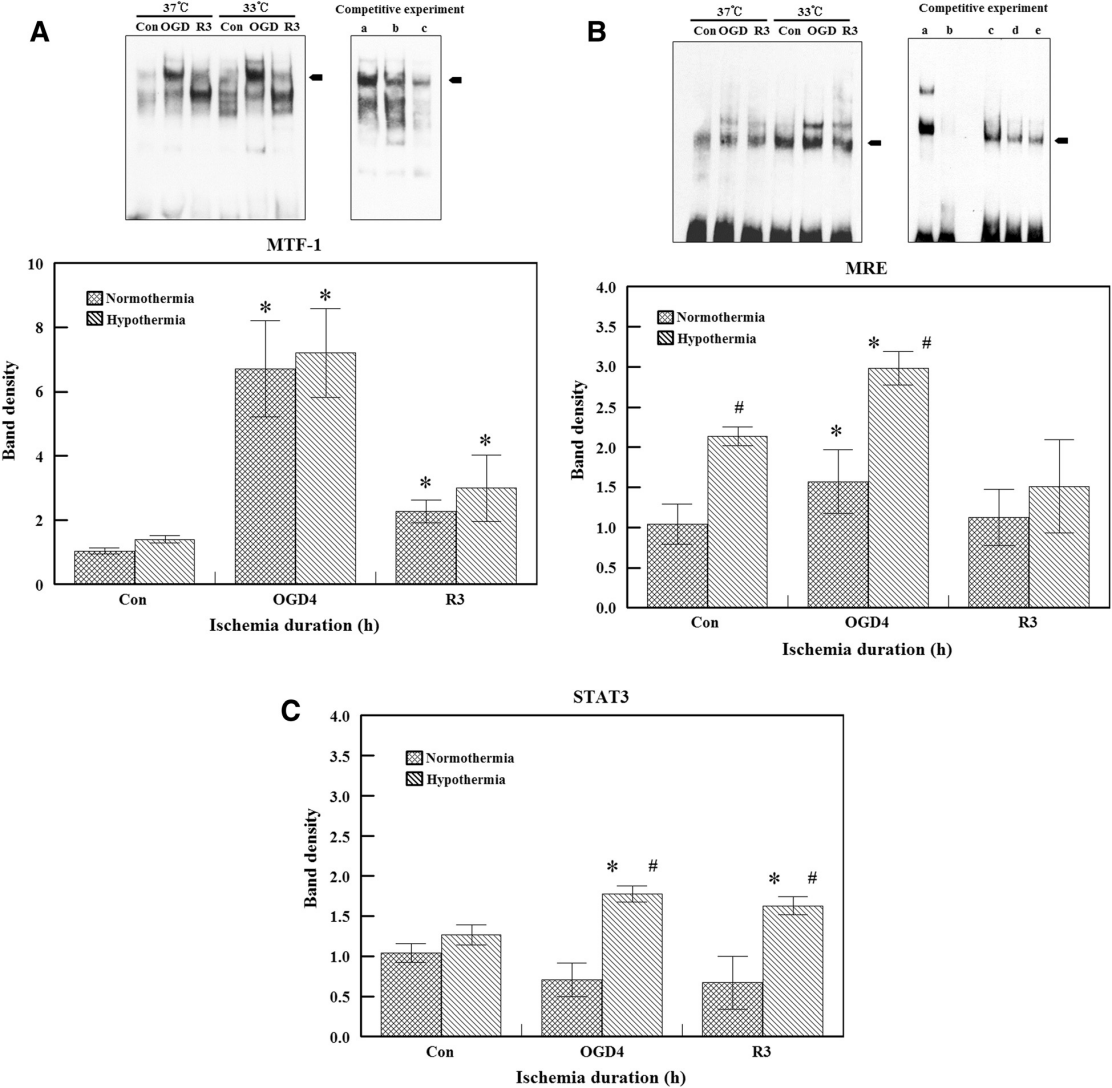
**Effects of ischemia or hypothermia on DNA binding activity. (A) **Electrophoretic mobility shift assay (EMSA) analysis of metal regulatory transcription factor 1 (MTF-1) or metal response elements d (MREd) binding activity was performed using nuclear extract proteins from the normal control, after 4 hours of oxygen glucose deprivation (OGD), and 3 hours after reperfusion at 37°C or 33°C. MTF1-DNA complexes were formed by incubating the biotin-labeled MREd probe with different nuclear extract proteins. The mixture was then separated in a non-denaturing polyacrylamide gel. The shifted bands (arrow) corresponding to the MTF-1/DNA complexes were visualized relative to the unbound double-stranded DNA. Competition experiment was performed as follows: a. OGD-stimulated sample with labeled MTF-1 probe. b. OGD-stimulated sample with cold and labeled MTF1 probe. c. Dexametasone-stimulated sample with labeled MTF-1 probe. DNA binding activity increased after OGD with both normothermia and hypothermia. DNA binding activity is expressed as mean ± S.E.M. of four experiments. *P <0.05 compared to the control. **(B) **EMSA analysis of MREa binding activity. A competitive experiment was performed as follows: a. Positive control with labeled control probe. b. Positive control with labeled probe and cold probe. c. OGD-stimulated sample with labeled MRE probe. d. OGD-stimulated sample with cold and labeled MRE probe. e. Dexametasone-stimulated sample with labeled MRE probe. MRE binding activity was increased by both ischemia and hypothermia. Binding activity is expressed as the mean ± S.E.M. of four experiments *P <0.05 compared to the control; #*P* <0.05 compared to normothermia. **(C) **STAT3 binding activity was measured using a STAT3 transcription factor assay kit. Hypothermia increased the binding of STAT3. Binding activity is expressed as the mean ± S.E.M. of four different experiments. *P <0.05 compared to the control; #*P *<0.05 compared to normothermia.

We determined whether the transcription factor MTF-1 is involved in the regulation of MT gene expression after ischemic insult or hypothermia. MTF-1 binds to a site present in the proximal promoter of the MT genes in response to intracellular heavy metal exposure [32,33], reactive oxygen species [34], and hypoxia [35]. MTF-1 binding to DNA was highly increased by 4 hours of OGD and returned to basal levels during reperfusion. Similar responses were observed with hypothermia (Figure 4A). The binding specificity was confirmed using unlabeled DNA probe or cold probe (Figure 4A, competition experiment b).

### DNA binding of MRE

In addition to MTF-1, we observed the binding activity of another MRE probe containing an MREa core sequence (5^′^-TGCGCCC-3^′^). The binding activity was increased after OGD exposure at 37°C. With hypothermia, MREa binding activity was significantly higher than that observed with normothermia both in the control and OGD groups (Figure 4B). Competitor MRE oligonucleotide confirmed the specificity (Figure 4B, competition experiment b).

### DNA binding activity of STAT3

STAT3 is a well-known transcription factor that binds to non-MRE sites of the MT gene promoter area. No significant change was found after ischemic insult at 37°C. However, OGD+R caused increased STAT3 binding in the hypothermic group (Figure 4C). Competition assays were performed in order to demonstrate the STAT3 binding specificity. HepG2 nuclear extract provided in the assay kit was used as a positive control for STAT activation. Competition assays were performed with the competitor oligonucleotide, a wild-type or mutated STAT consensus binding site (data not shown).

### STAT3 phosphorylation

**Figure 5:**
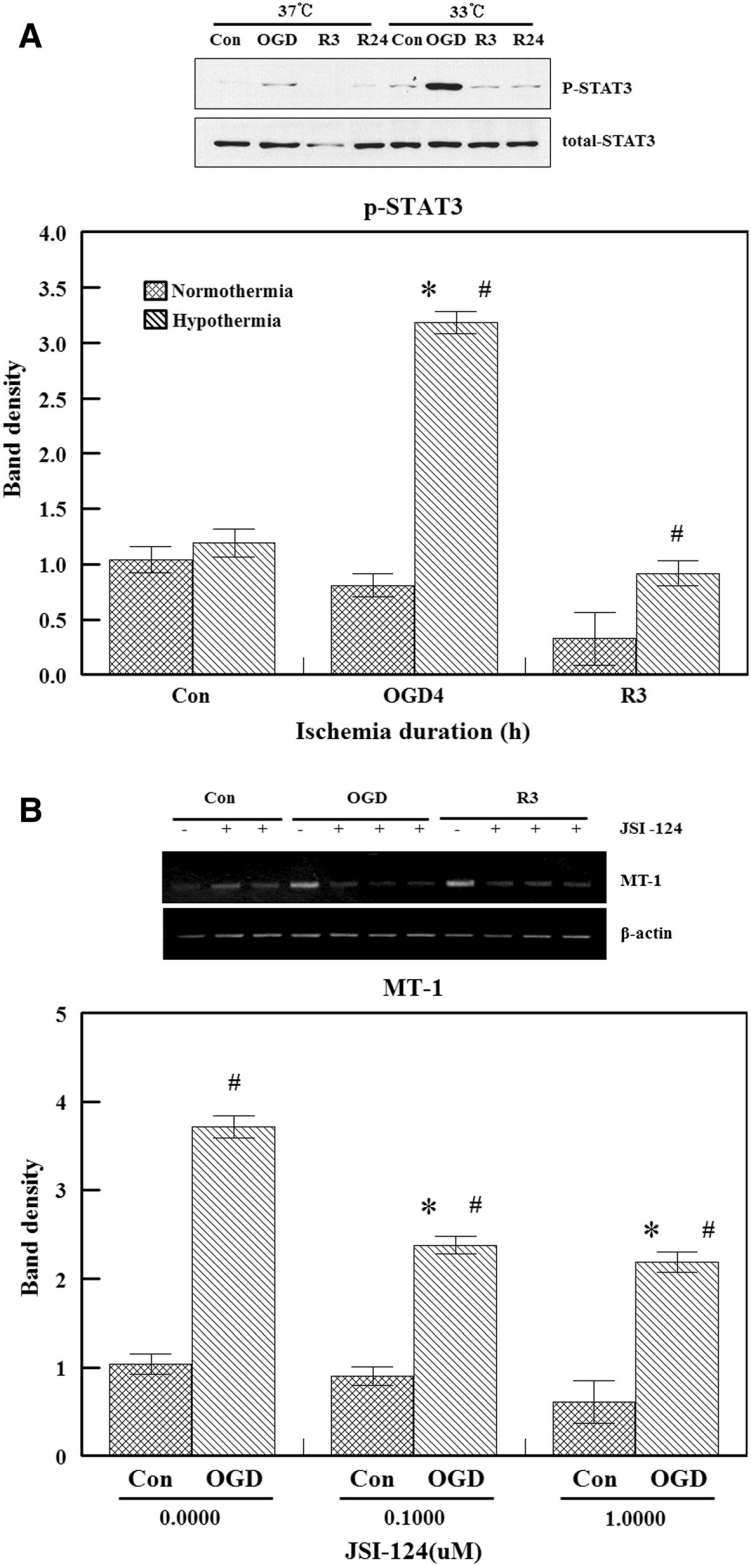
**Effect of hypothermia on signal transducer and activator of transcription 3 (STAT3) phosphorylation and effect of a STAT3 inhibitor. (A) **Western blot analysis of phosphorylated STAT3 (Ser727) was performed. After 4 hours of oxygen glucose deprivation (OGD), phosphorylation reached a peak with hypothermia. Relative levels of phosphorylated STAT3 are expressed as the mean ± S.E.M. of three experiments. *P <0.05 compared to the control; #*P *<0.05 compared to normothermia. **(B) **bEnd.3 cells were treated with either vehicle or JSI-124 (0.1 and 1 μM) 30 minutes before OGD. The expression of metallothionein (MT) mRNA was analyzed by reverse transcription polymerase chain reaction (RT-PCR). JSI-124 inhibited the induction of MT expression. Values represent the mean ± S.E.M. of three different experiments. *P <0.05 compared to the control; #*P *<0.05 compared to normothermia.

It is known that phosphorylated STAT3 binds to gene promoters and regulates the transcriptional activity of target genes. It has also been reported that increased STAT3 phosphorylation is accompanied by increased synthesis of MT in the presence of myocardial ischemia reperfusion injury [36]. In the present study, STAT3 phosphorylation (Ser 727) was measured using nuclear extracts from bEnd.3 cells. Western blot analysis demonstrated that STAT3 phosphorylation was significantly increased after 4 hours of OGD with hypothermia while no change in the level of phosphorylated STAT3 was observed under normothermic conditions (Figure 5A). This increase of STAT3 phosphorylation concurs with the binding activity assay data and implies that STAT3 plays a temperature-dependent role in the regulation of MT gene transcription.

### Effect of STAT3 inhibition

To confirm whether the STAT3 signaling pathway influences MT gene expression, we used JSI-124 (also known as cucurbitacin I) as a STAT3 inhibitor. JSI-124 is a potent and highly selective inhibitor of the JAK/STAT3 pathway [37]. MT-I gene expression was increased by OGD+R while treatment with JSI-124 (0.1 and 1 μM) significantly reduced the level of MT gene expression (Figure 5B). Neither U0126 (an ERK inhibitor) or SB203580 (a JNK inhibitor) affected MT transcription (data not shown).

### Effect of DNA demethylation on MT gene expression

**Figure 6:**
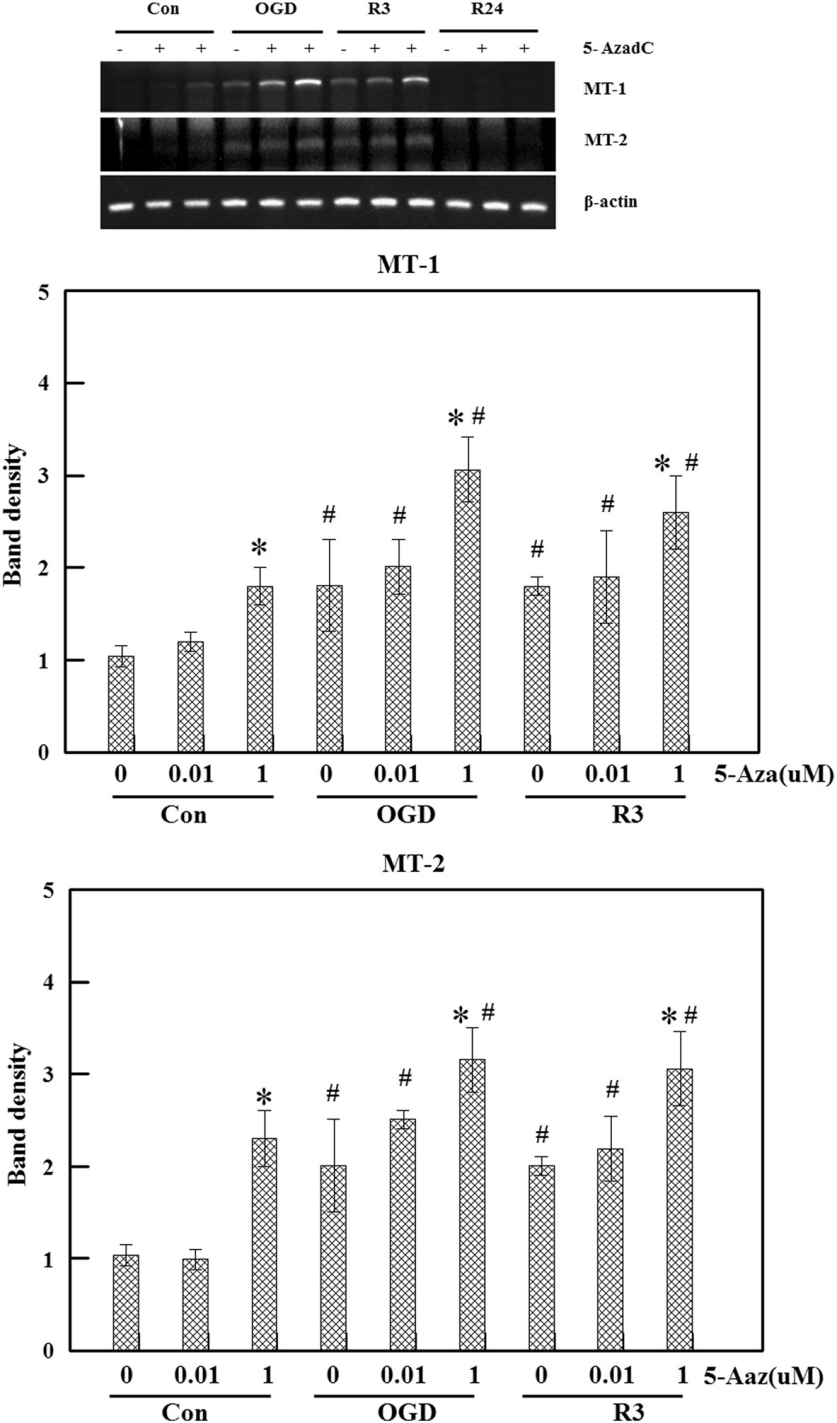
**Effect of 5-Aza treatment. **Cells were treated with 5-Aza (0.01, and 1 μM) for 3 days and then subjected to oxygen glucose deprivation and reperfusion (OGD+R). RNA extracted from the cells was used for reverse transcription polymerase chain reaction (RT-PCR). Metallothionein (MT) induction was potentiated by 5-Aza treatment in a dose-dependent manner. Values represent the mean ± S.E.M. of three different experiments. *P <0.05 compared to 0 uM of 5 Aza; #*P *<0.05 compared to the control group.

DNA methylation at position 5 of the cytosine in CpG dinucleotides is the most significant epigenetic mechanism that leads to altered transcription of target genes [31]. It was also reported that MT-1 promoter methylation causes MT gene silencing in cancer cells [38,39]. Thus, methylation of the MT promoter region may be related to gene expression under various conditions such as ischemia or hypothermia. We first treated bEnd.3 cells with 5-Aza, a demethylating agent, for 3 days to reduce gene methylation. The cells were then exposed to OGD+R and mRNA levels were measured with RT-PCR. MT expression was barely detected in the control group. Treatment with 5-Aza (1 μM) increased in MT levels even without OGD+R. Furthermore, 5-Aza treatment increased the induction of MT gene expression in a dose-dependent manner after OGD+R (Figure 6).

### Methylation profiles of the MT promoter region

**Figure 7:**
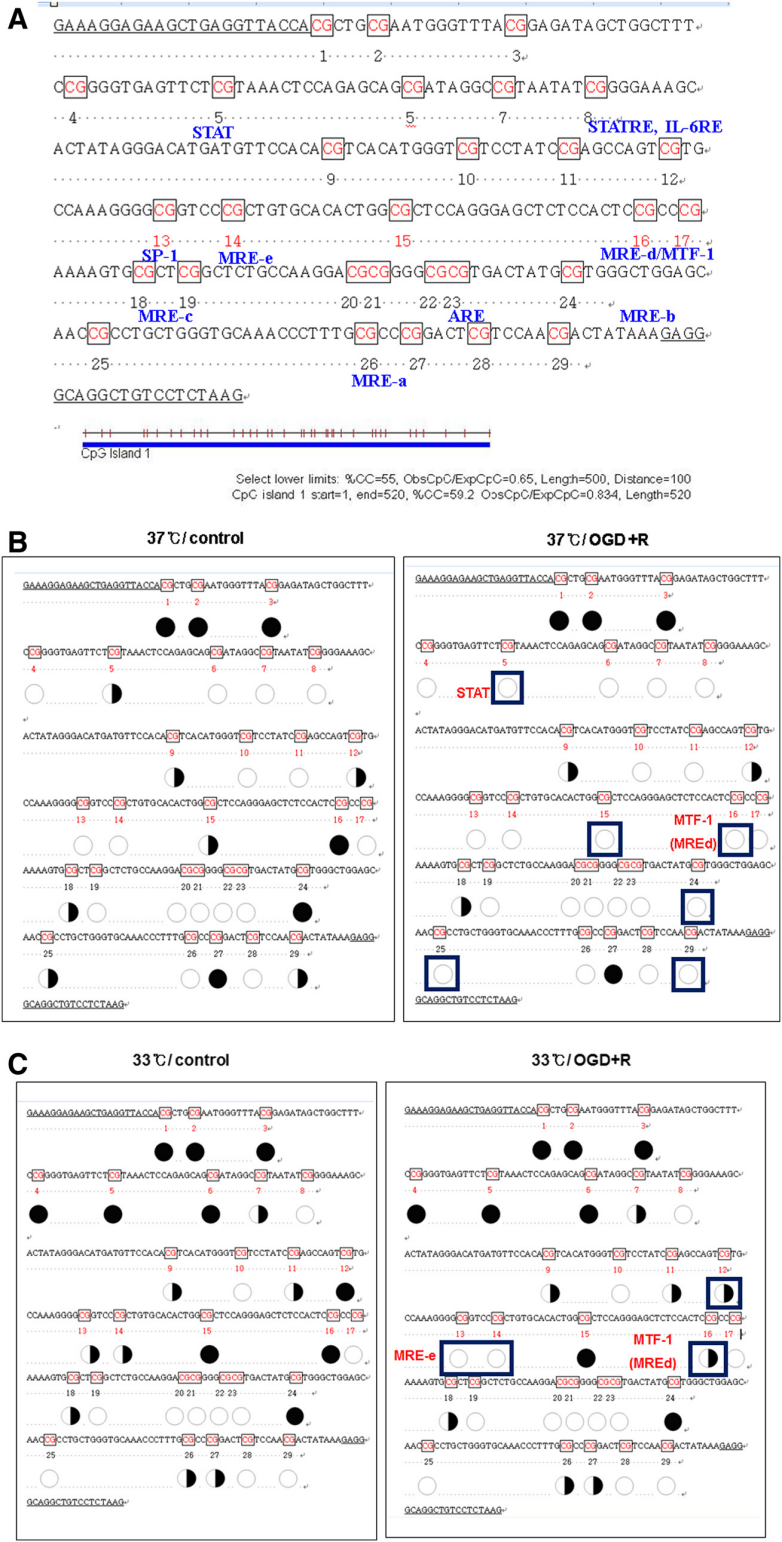
**Effect of ischemia or hypothermia on methylation profiles of the 5**^**′**^**-regulatory region of the metallothionein 1 (MT1) promoter. (A) **Partial sequences of the 5^′^-regulatory region of the mouse MT1 is based on Gene Bank nucleotide sequence [GenBank: J00605]. Twenty-nine CpG sites are marked in red. Some transcriptionally active binding elements such as metal response elements (MRE), signal transducer and activator of transcription (STAT), and antioxidant response elements (ARE) are shown in blue. **(B) **Methylation profiles of the normothermia control and normothermia oxygen glucose deprivation and reperfusion (OGD+R) were compared. **(C) **Methylation profiles of the hypothermia control and hypothermia OGD+R were compared. Bisulfite pyrosequencing analysis was performed for 29 CpG sites near the transcription initiation site of the 5^′^-regulatory region. Methylated status of the CpG sites is expressed as an open circle (○) for unmethylated and a black circle (●) for methylated.

Since our data suggested that MT gene transcription is affected by DNA methylation under normal or OGD+R conditions, we determined the precise methylation status of putative CpG sites in the MT promoter area. After bisulfite treatment, pyrosequencing was performed on genomic DNA isolated from bEnd.3 cells. The sequence analyzed in this study corresponded to the proximal promoter region spanning from -300 to +1 of the mouse MT-1 gene. Sequence analyses of the selected region revealed 29 CpG sites and some of those sites overlapped the transcription factor binding sites (Figure 7A).

Under normothermic conditions, there were six CpG sites, demonstrating differences in methylation profiles observed under control and OGD+R conditions (Figure 7B). Among these six sites, the STAT and MTF-1 binding sites or MREd were known to be transcription factor binding areas. As shown in Figure 7B, CpG islands were in a semi-methylated state under normal conditions while these islands in cells stimulated with OGD+R were more demethylated. The overall percentages of methylation were 62.4 ± 7.1% (control at 37°C), 50.5 ± 5.3% (OGD at 37°C), and 38.4 ± 3.4% (OGD+R at 37°C). Both STAT and MTF-1 binding sites are more demethylated after OGD+R (Figure 7B).

With hypothermia, there were four CpG sites, demonstrating differences in methylation profiles observed during the control and OGd+R conditions (Figure 7C). Among these four sites, MREe and MTF-1 binding sites or MREd were known to be transcription factor binding area. In Figure 7C, CpG islands were in a semi-methylated state under normal conditions while those in cells stimulated with OGD+R were more demethylated. The overall percentages of methylation (Figure 7C) were 45.6 ± 10.1 (control at 33°C), 38.5 ± 6.2% (OGD at 33°C), and 39.7 ± 4.2% (OGD+R at 33°C). Both the MREe and MTF-1 binding sites are more demethylated after OGD+R. Demethylated transcription binding sites following OGD+R might be associated with the induction of MT expression and increased transcription factor binding activities.

When the normothermic control group was compared to the hypothermic control group, 13 CpG sites were found, indicating different methylation profiles. Among these 13 sites, ten sites were more methylated and three were less methylated in the hypothermic group. When the normothermic OGD+R group was compared to the hypothermic OGD+R group, ten CpG sites were found, demonstrating differences in methylation profiles. Among these ten sites, nine sites were more methylated and one site was less methylated in the hypothermic group. These findings indicated that hypothermia seems to shift the MT promoter region to a more methylated state.

### Effect of ischemia or hypothermia on DNA methyltransferases and methyl-CpG binding domain proteins

**Figure 8:**
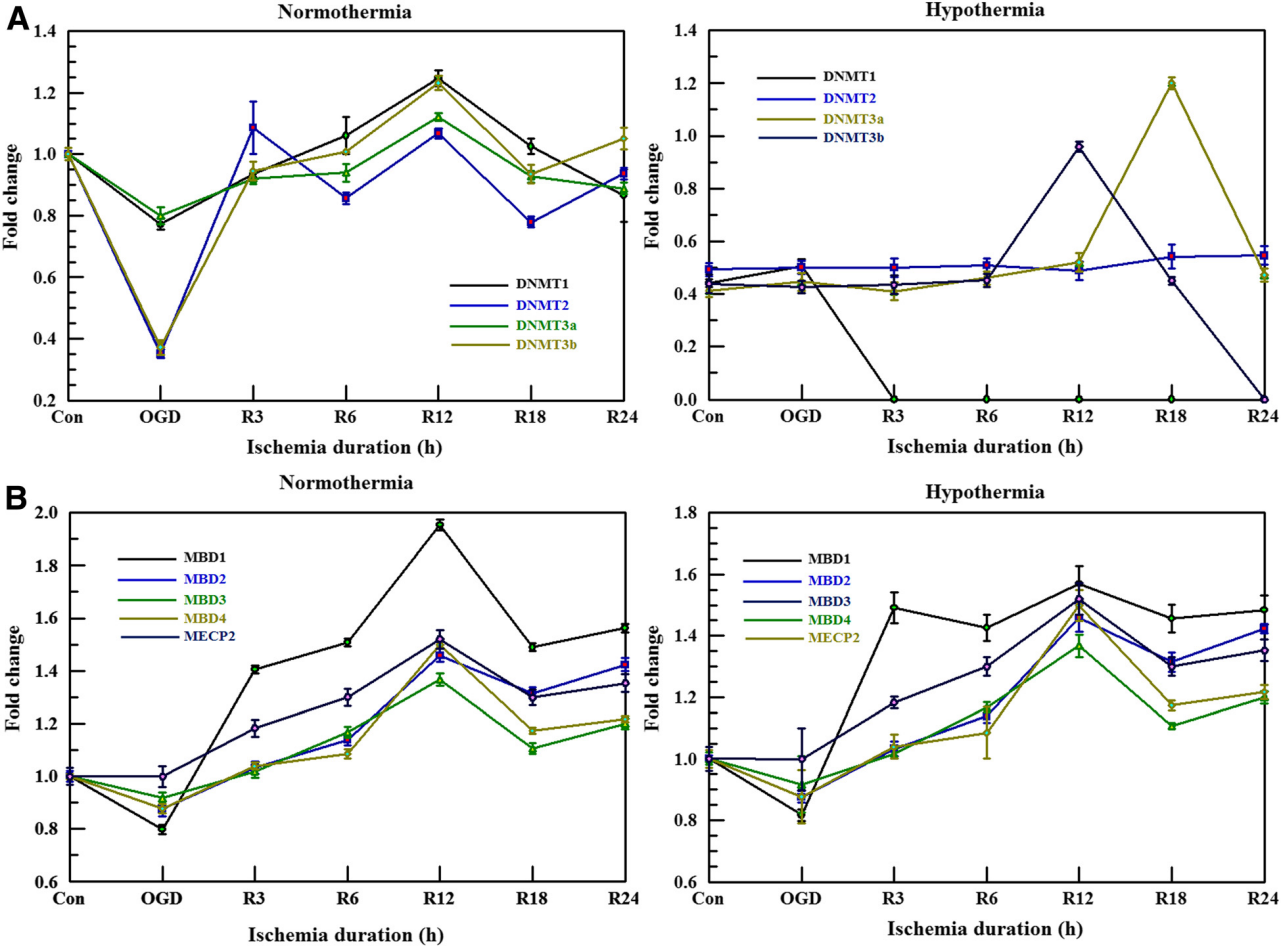
**Effect of ischemia or hypothermia on methylation regulating factors (A). **We measured the mRNA levels of methylation related proteins such as DNA methyltransferases (DNMTs) 1, 2, 3, and 4 using real-time PCR. RNA was harvested from cells exposed to 4 hours of oxygen glucose deprivation (OGD) and to reperfusion for 3 to 24 hours. **(B)** The levels of methyl-CpG binding domain proteins (MBDs) 1, 2, 3, and 4 along with methyl-CpG binding protein-2 (MECP-2) were measured using the same samples and analysis tools as in 6A. Values represent the mean ± S.E.M. of three different experiments.

Since methylation can interfere with the regulation of MT gene expression, we measured the mRNA levels of methylation-related proteins including DNA methyltransferases (DNMTs) 1, 2, 3, and 4 (Figure 8A) along with methyl-CpG binding domain proteins (MBDs) 1, 2, 3, and 4 and methyl-CpG binding protein-2 (MECP-2) using real-time PCR (Figure 8B). In general, the levels of DNMTs were lower with hypothermia and DNMT1 expression was markedly decreased by OGD+R (Figure 8A).

## Discussion

In the present study, we demonstrated that the expression of MT genes was induced by hypothermia under ischemic conditions. Both mRNA and protein levels were increased by OGD+R and hypothermia. Since the induction of MT expression was observed by hypothermia without ODG+R in our unpublished experiment, both ischemia and hypothermia seem to be triggers of MT gene expression. Given that many studies and our own data provided evidence of the protective effect of MT in brain injury models [14,40,41], we suggest that MT is a novel factor that contributes to hypothermic protection.

To further elucidate the molecular mechanism underlying MT induction by ischemia or hypothermia, we evaluated some transcriptional factors and their binding elements on the MT promoter area. When binding activities of the transcription factors to the MT promoter area were measured, MTF-1/MREd showed strong activity after OGD. STAT3 activity was greater with hypothermia while that of MREa was increased by both hypothermia and OGD. STAT3 phosphorylation after OGD was enhanced by hypothermia and inhibition of the STAT3 pathway reduced MT expression. According to other reports, MT expression is regulated by several promoter regions including MREs, antioxidant response elements (AREs), and inteleukin-6 responsive elements [17,18,21,35]. The MRE motif is a major site for MTF-1 binding induced by metal ions and other stimuli [18,21,35]. The mouse MT promoter region contains six MRE sequences. The MREd sequence is the strongest among the six MREs [23] and MREa is most active in response to zinc [42]. Stuart *et al*. [24] showed that the inactivation of the internal MREs within direct MRE repeats leads to the loss of promoter activity, suggesting that every MRE copy within the repeats participates in cooperative rather than independent action. Additionally, multiple MREs scattered throughout the promoter region might synergistically interact [42]. Based on our data and that from others reports, we expect that MT gene regulation is dependent on hypothermia, ischemia and different types of transcription factors or that binding elements are synergistically involved depending on the type of stimuli.

In addition to MREs, STATs have been shown to induce MT transcription and the consensus STAT sequence (TTN5AA) is present in the MT-I promoter [25]. STAT activation plays an important role in mediating transcriptional regulation by cytokines, hormones, and growth factors [36,43,44]. However, STAT activation by hypothermia has not been clearly demonstrated. STATs are activated by phosphorylation on conserved tyrosine and serine residues. Whereas phosphorylation of STATs on tyrosine residues regulates dimerization, serine/threonine phosphorylation is believed to influence nuclear translocation and DNA binding activity [45]. As expected, we demonstrated that hypothermia increased STAT3 binding activity and serine 727 phosphorylation. However, STAT3 binding activity was not altered in the normothermic group. Our results are consistent with those from other reports showing that STAT binding activity does not change after ischemia or reperfusion in a rat middle cerebral artery occlusion model [16]. Nevertheless, one *in vivo* study found that STAT3 activation protects the myocardium from ischemia-reperfusion injury through MT [46]. We are not certain whether this discrepancy is due to differences between cell types or other factors. In the present study, JSI-124 treatment significantly suppressed the induction of MT expression. This inhibitor is known to exert an inhibitory effect on STAT3 DNA binding and STAT3 mediated gene transcription [37]. Blaskovich *et al*. [37] demonstrated that JSI-124 is highly selective for JAK/STAT3 and does not affect other pathways such as those mediated by Ras, Akt, Erk1/2, or JNK. When cells were treated with U0126 (a MEK1/2 inhibitor) or SB203580 (a JNK inhibitor), no effect on MT induction was observed. We therefore concluded that that the action of JSI-124 we observed is specific for the STAT3 pathway.

DNA methylation is a major mechanism that regulates cell-specific gene expression since CpG methylation in the promoter region abrogates transcription factor binding [47]. It is known that chromatin conformation mediates the accessibility of DNA elements to transactivators such as transcription factors and is critical for the methylation-dependent transcriptional inhibition. Given that the epigenetic regulation of MT gene expression has been reported [30,31], we investigated the possibility of methylation-dependent regulation under our experimental conditions by treatment with 5-Aza. Since MT gene expression was induced by 5-Aza, we hypothesized that MT gene expression during ischemia or hypothermia is related to DNA methylation in the MT promoter region. By performing bisulfite pyrosequencing of the MT-I gene promoter region, we obtained detailed methylation profiles.

After ischemic insult was applied, a reduced methylated or under-methylated state was observed regardless of temperature. This suggests that ischemic stimuli activate many genes through release of methylation-dependent transcriptional inhibition factors as we expected. We can easily hypothesize that hypomethylation will lead to enhanced expression of the MT gene similar to that found after ischemic insult. When we inspected the methylation profile associated with hypothermia, the CpG sites in the MT promoter region after hypothermia were more methylated compared to normothermic conditions. Only the MREa site was in a reduced methylation state after hypothermia. Even though the general methylation profile was not consistent with MT induction, the MREa methylation state concurred with binding activity assay results and induced MT expression.

Contrary to our expectations, the STAT area was more methylated with hypothermia. One study reported that demethylation of 10 to 20% of the CpG islands in the promoter is adequate to induce MT-I gene expression with cadmium or zinc in mouse lymphosarcoma cells [48]. It has also been shown that MTF-1 binding to MREa is affected by methylation status, but in the case of MREd this is unaffected by DNA methylation *in vitro*[49]. Therefore, it is likely that MTF-1 binding to MREs is differentially modulated by methylation status. From these data, we conclude that although DNA methylation plays a key role in gene transcription, CpG methylation alone was not sufficient to regulate MT gene transcription in our experimental model. It seems that both promoter methylation and transcription factor activity contribute to the regulation of gene expression.

Our study provides evidence of methylation-dependent gene regulation following ischemia or hypothermia. Since both stimuli were applied for short periods of time (within the range of hours), we suggest that epigenetic control by methylation can be achieved even in short-term conditions in addition to long-term conditions. To identify the causes of methylation profile changes by ischemia or hypothermia, we observed alterations of some methylation regulating factors such as DNMTs and methyl-CpG binding proteins including MBDs or MECP-2. The levels of DNMTs were generally lower with hypothermia. In particular, DNMT1 expression was markedly decreased by OGD+R with hypothermia. Since DNMT1 is the most abundant DNA methyltransferase and considered to be the key methyltransferase in mammalian cells, lower levels of DNMT1 might be related with changes in the demethylation state observed under OGD+R conditions. Even though we could not clearly identify correlations between changes in the levels of these proteins and methylation patterns, we expect that more information will be obtained in upcoming studies.

## Conclusions

In summary, we demonstrated that MT expression was markedly increased under hypothermic conditions using an *in vitro* ischemia model. Our findings can explain, at least in part, the protective role of hypothermia. The induction of MT expression was mediated by altered binding activity of transcription factors such as MTF-1 and STAT3. In addition, DNA methylation contributed to MT gene regulation. Our findings provide new insight for understanding the molecular mechanisms underlying the ability of hypothermia to protect against ischemia.

## Abbreviations

AREs: antioxidant response elements; CNS: central nervous system; DNMTs: DNA methyltransferases; EMSA: electrophoretic mobility shift assay; GREs: glucocorticoid response elements; LDH: lactate dehydrogenase; LPS: lipopolysaccharide; MECP-2: methyl-CpG binding protein-2; MREs: metal response elements; MT: metallothionein; MTF-1: metal regulatory transcription factor 1; OGD: oxygen glucose deprivation; OGD+R: oxygen glucose deprivation and reperfusion; RT-PCR: reverse transcription polymerase chain reaction; STAT3: signal transducer and activator of transcription 3; 5-Aza: 5-aza-2^′^-deoxycytidine.

## Competing interests

The authors declare that they have no competing interests.

## Authors’ contributions

HSH and JKK were responsible for the foundation, conception, and design of the experiment; analysis of experimental data and supervising the manuscript writing. YHP was the major participant in conducing the experiments, data acquisition, statistical analysis, and manuscript writing. YML, DSK, JCP, and KHS participated in performing the experiments as well as data acquisition and analysis. All authors read and approved the final manuscript.
